# Perspective Overview of Changing Population Immunity to COVID-19 in the Context of Infection, Vaccination, and Emerging SARS-CoV-2 Variants

**DOI:** 10.3390/pathogens14121197

**Published:** 2025-11-24

**Authors:** Ranjan Ramasamy

**Affiliations:** ID-FISH Technology, 556 Gibraltar Drive, Milpitas, CA 95035, USA; rramasamy@idfishtechnology.com

**Keywords:** COVID-19, immune evasion by SARS-CoV-2, innate and adaptive immune responses to SARS-CoV-2, population immunity to COVID-19, SARS-CoV-2 evolution, SARS-CoV-2 variants

## Abstract

The changing state of protective immunity to COVID-19 in the global population in the six years since COVID-19’s origin in 2019 is examined in the context of the (i) circulation of SARS-CoV-2 in the population, (ii) widespread use of different types of COVID-19 vaccines beginning in December 2020 and continuing to the present time, and (iii) ongoing evolution of SARS-CoV-2 to produce mutant viruses with greater infectivity, replication rate, evasion of immunity, and transmissibility. The outlook, and possible vaccine strategies, for the future control of COVID-19 are also examined.

## 1. Background

### 1.1. Severe Acute Respiratory Syndrome Coronavirus 2 (SARS-CoV-2) in the Context of Other Human Coronaviruses

Coronavirus disease 2019 (COVID-19) was first identified in December 2019 in Wuhan, China, and the responsible pathogen characterized and named as the severe acute respiratory syndrome coronavirus 2 (SARS-CoV-2). SARS-CoV-2 is closely related to two other highly pathogenic betacoronaviruses of zoonotic origin, SARS-CoV-1 and MERS-CoV. It is also related to several widely circulating and less pathogenic human coronaviruses, HCoV-229E, HCoV-NL63, HCoV-OC43, and HCoV-HKU1, that mostly cause mild infections in the upper respiratory tract (URT) and 10–30% of common colds [1]. SARS-CoV-2 is a membrane-enveloped virus with a 30 kb positive-sense RNA genome that codes for 29 viral proteins [1].

### 1.2. Functions of Key SARS-CoV-2 and Host Cell Proteins in the Initial Infection of Human Respiratory Tract Epithelial Cells

The spike glycoprotein (S) is present as a trimer on the membrane envelope of SARS-CoV-2 and is formed from a 1273 amino acid monomer protein (GenBank QIH45093). S contains a receptor-binding domain (RBD) in its N-terminal S1 domain that binds to the angiotensin-converting enzyme 2 (ACE2) receptor on the epithelial cells of the respiratory tract, and a fusogenic peptide in the C-terminal S2 region that subsequently helps fuse the virus and host cell membranes to permit entry of viral RNA into the cytoplasm [2,3]. Two human cell proteases, furin and a transmembrane serine protease (TMPRSS2), are, respectively, important for cleaving the S protein at the S1/S2 junction to facilitate ACE2 binding, and within the S2 to expose a hydrophobic region that allows the fusion of the virus and host cell membranes [3]. SARS-CoV-2 can also enter the cytoplasm through endocytosis followed by a protease-dependent S-mediated fusion of the membranes of the endosome and virus [3]. The S protein in the plasma membrane of virus-infected respiratory tract cells also facilitates the fusion of the membrane of virus-infected cells with the those of adjacent uninfected cells, creating syncytia that further facilitate the spread of the SARS-CoV-2 infection [3].

### 1.3. Acquisition of SARS-CoV-2 Infections

SARS-CoV-2, like other respiratory viruses, can be transmitted by direct or fomite contact with the mucous membranes of the eye, nose, and mouth. However, inhalation of SARS-CoV-2 virions that are exhaled in the breath or through coughing and sneezing of persons with COVID-19 is now recognized to be the dominant method of infection [4,5,6,7]. Like influenza A [8], there is an upsurge of SARS-CoV-2 infections during cold winter seasons in temperate zone climates [9,10]. This has been largely attributed to weaker innate and adaptive immune responses to SARS-CoV-2 in the URT when inhaled air in winter is inadequately warmed and humified in the URT [4,5,6]. However, the transmission of SARS-CoV-2 can also increase when people venture outdoors more in warm weather and when new virus variants with greater fitness emerge, so that COVID-19 prevalence can sometimes also surge during other seasons [9,10].

### 1.4. Pathology and Prevalence of COVID-19

Studies conducted during the early stages of the COVID-19 pandemic showed that healthy young individuals infected with SARS-CoV-2 often develop a mild or asymptomatic form of the disease because they can rapidly eliminate the virus from the URT through an effective URT immune response [6,11]. The different types of innate [6] and adaptive [6,11] immune responses that protect against infection of the URT by SARS-CoV-2 and human genetic factors that influence protective immunity have been previously described. The infection of the lower respiratory tract (LRT) occurs when the virus is not eliminated in the URT [4,5,6], resulting in severe pneumonia in about 15% of non-immune patients and an acute respiratory distress syndrome, which is difficult to treat, in about 5% of non-immune patients [12,13]. Persons with underlying deficiencies in their immune system and the elderly are particularly susceptible to developing severe disease, hospitalization, and death (7). Dysfunctional immune responses in the LRT and systemic spread of the virus are also characteristics of severe COVID-19 [12,13,14]. The World Health Organization (WHO) recorded 771 million confirmed cases of COVID-19 and 7 million resulting deaths worldwide as of 4 October 2023 [15].

The WHO first declared COVID-19 to be a Public Health Emergency of International Concern (PHEIC) on 30 January 2020 [16], and then a pandemic on 11 March 2020 [17]. However, the rapid decline in morbidity and mortality after COVID-19 vaccination was introduced in December 2020, led to the WHO declaring an end to the PHEIC on 5 May 2023 [18]. After May 2023, the WHO has continued to monitor the global COVID-19 situation, reporting rolling 28-day estimates of worldwide COVID-19 cases and mortality [19]. There were 114,882 confirmed cases and 1494 deaths due to COVID-19 globally from 11 August 2025 to 07 September 2025 recorded by the WHO, but these numbers may be underestimates because of the variable monitoring and reporting of COVID-19 from different countries [19].

### 1.5. The Impact of Vaccination on Mortality and Morbidity During the COVID-19 Pandemic

The negligible pre-existing immunity against SARS-CoV-2 in the global population, and the high morbidity and mortality associated with COVID-19 during the early stage of the pandemic, led to the rapid development of vaccines against COVID-19 and their deployment beginning in December 2020 [20]. The early COVID-19 vaccines were based on delivery platforms used for vaccines against other diseases that were quickly adapted for delivering SARS-CoV-2 immunogens. Such vaccine platforms included messenger RNA, DNA, protein subunits, viral vectors, inactivated whole SARS-CoV-2, and virus-like particles [7,21,22,23]. Inactivated whole-virus vaccines, as well as mRNA and nonreplicating adenovirus-vectored vaccines expressing S, all of which were administered by intramuscular injection, were the principal types of COVID-19 vaccines that were rapidly approved for use in many countries [21,22]. Such vaccines generated sufficient LRT and systemic immunity to significantly protect against severe COVID-19 and systemically disseminated virus infections, but a review of the then available experimental data concluded that their ability to prevent initial infection of the URT and the transmission of SARS-CoV-2 from infected to uninfected persons were less than desirable [20,23,24]. More rapid elicitation of protective immune response in the URT to combat early infection with SARS-CoV-2 was considered needed to achieve this [20,24]. Efforts to develop safe and effective mucosal COVID-19 vaccines to achieve this objective are therefore presently under way, and these include many candidate intranasally administered vaccines, some of which are already in clinical trials [20,24,25,26].

The administration of COVID-19 vaccines to a large proportion of the population in many countries from December 2020 onwards enhanced population immunity and reduced severe COVID-19, as well as the associated hospitalizations and mortality. The impact of COVID-19 vaccination on mortality and morbidity is best assessed during the early vaccine roll-out period because this minimizes the effects of immunity induced through infection. In the first year of the COVID-19 vaccination, from December 2020 to December 2021, 55.9% of the global population were estimated to have received one dose of a vaccine, 45.5% to have received two doses, and 4.3% a third booster immunization [27]. During this first year of vaccination, 14.4 million (95% confidence interval or CI of 13.7–15.9 million) officially reported COVID-19 deaths, and 19.8 million (95% CI 19.1–20.4 million) deaths from the more reliable excess mortality data, were estimated to have been averted by vaccination in 185 countries [27]. Vaccination at this early stage of the pandemic also reduced SARS-CoV-2 infections and hospitalizations. In total, 8 million fewer COVID-19 infections, 0.7 million fewer hospitalizations, and 0.12 million less deaths in the first six months of COVID-19 vaccine deployment from 14 December 2020 to 3 June 2021 were attributed to vaccination against COVID-19 in the US [28]. Data from Israel over the first 112 days of an mRNA vaccine use from 20 December 2020 to 10 April 2021 were equally impressive [29]. Among persons aged >16 y who received at least the priming vaccine or the priming vaccine followed by a booster 21 d later, 158,665 COVID-19 infections (95% CI of 144,640–172,690), 24,597 hospitalizations (95% CI 18,942–30,252), and 5532 deaths (95% CI 3085–7982) were estimated to have been averted in Israel [29]. Protection conferred by the vaccination was particularly pronounced in elderly Israelis aged >65 y [29]. Without vaccination during this stage of the COVID-19 pandemic, a 300% increase in hospitalizations and deaths, which would have overwhelmed the healthcare system in Israel, were predicted to occur [29].

### 1.6. Origin, Evolution, and Spread of Early SARS-CoV-2 Variants

During the early stage of the COVID-19 pandemic before the widespread use of vaccines, selection for genetic changes that resulted in greater infectivity and higher virus replication rates within infected cells, together with enhanced disease severity and transmission, characterized emergent SARS-CoV-2 variants [7,30]. The variant that arose in March 2020 carrying a D614G mutation in S and a linked mutation in the RNA-dependent RNA polymerase of the virus was an example of this process [30]. The Alpha variant that arose in mid-2020 was another, but Alpha carried additional mutations in S that reduced neutralization of the virus by antibodies [30]. The Delta variant that spread from April 2021 onward caused more severe disease than Alpha and showed even greater reduction in its ability to bind neutralizing antibodies [30]. After widespread infection and vaccination, SARS-CoV-2 variants that were more successful in evading antigen-specific adaptive immunity but that caused no greater disease severity began to emerge, e.g., Omicron in November 2021 [30]. The molecular immunological and virological factors underlying the origin and global spread of these important SARS-CoV-2 variants during the pandemic period up to May 2023 have been reviewed [30].

Meta-analysis suggested that the progressively greater evasion of immunity by variants evolving with the increasing use of COVID-19 vaccines, and the spread of SARS-CoV-2 infections were associated with the reduced effectiveness of vaccines based on the ancestral Wuhan strain S proteins in preventing both infection and severe disease [31]. The understanding of the immunological and protein-related structural basis of how the various mutations in S reduce antibody-mediated neutralization and influence binding to ACE2 receptors has advanced rapidly [32,33,34]. Besides the evidence supporting the greater evasion of antibody-mediated invasion inhibition [35,36] and more efficient utilization of TMPRSS2 for invasion [35], limited evidence suggests that the greater prevalence of the JN.1 Omicron variant in 2023/4 may also have been due to mutations in S and other viral proteins that reduced recognition by cytotoxic T cells [36].

Increased viral fitness, determined by infectivity, replication rate, transmission, and immune evasion, is the force driving the selection of SARS-CoV-2 variants that replace previous less competitive virus strains. The SARS-CoV-2 genome encodes a 3′ to 5′ proofreading exonuclease that excises mismatched nucleotides during replication. However, a spontaneous mutation rate of 1–5 × 10^−6^ nucleotides per virus replication cycle has been estimated from in vitro experiments [37] and these mutations provide the basis for the selection process. Chronic SARS-CoV-2 infections that occur in patients with weak immunity and those undergoing treatment with anti-viral chemotherapeutics have therefore been proposed to facilitate the generation of SARS-CoV-2 variants [30].

The continuing evolution of SARS-CoV-2 variants globally and their deposited genomic sequences are analyzed and made publicly available by the Global Initiative on Sharing All Influenza Data (GISAID) [38]. Each major variant identified since the original Wuhan-Hu-1 strain in 2019 has, in turn, generated multiple other variants [38]. Some variants became prominent in localized areas, while others spread widely across the world. The early variants have now been replaced by more recent variants of the Omicron lineage [38]. A few variants were designated as variants of concern (VOCs) by the WHO because they displayed one or more of the following features compared to other variants: (i) a detrimental change in clinical disease severity; (ii) a change in COVID-19 epidemiology, causing substantial impact on the ability of health systems to provide care to patients with COVID-19 and other illnesses and therefore requiring major public health interventions; or (iii) a significant decrease in the effectiveness of available vaccines to protect against severe disease [39]. Since 2023, the WHO has designated VOCs only with Greek letters and after May 2023 this has only been applied to Omicron [39]. Omicron has since given rise to numerous variants, of which some have been termed variants of interest (VOIs) or variants under monitoring (VUMs) [39]. A VOI is a SARS-CoV-2 variant (i) with genetic changes that are predicted or known to affect virus characteristics such as transmissibility, virulence, antibody evasion, susceptibility to therapeutics, and detectability, and (ii) that is identified to have a growth advantage over other circulating variants in more than one WHO region with increasing relative prevalence alongside an increasing number of cases over time, or other apparent epidemiological impacts to suggest an emerging risk to global public health. A VUM is a SARS-CoV-2 variant with genetic changes that are suspected to affect virus characteristics together with early signals of growth advantage relative to other circulating variants (e.g., growth advantage which can occur globally or in only one WHO region), but for which evidence of phenotypic or epidemiological impact remains unclear, requiring enhanced monitoring and reassessment pending new evidence. If a variant has an unusually large number of mutations in known antigenic sites but very few sequences, and it is not possible to estimate its relative growth advantage, such a variant can be designated a VUM, if there is also evidence of community transmission in ≥two countries within a 2–4-week period. VOCs, VOIs, and VUMs require different actions by the WHO and health ministries of member countries [39]. SARS-CoV-2 variants are presently classified by the use of Greek letters, Nextclade (year and order of assignment), or Pango (alphabetical prefix followed by a numerical suffix) nomenclatures [30,38,39,40].

## 2. COVID-19 Prevalence and Vaccination Status in 2025

Table 1 shows that in comparison with the 771 million confirmed cases of COVID-19 and 7 million deaths recorded by the WHO until 4 October 2023 [15], there was an increase in the corresponding numbers recorded by the WHO on 14 September 2025 [19]. The WHO rolling data also showed that the increase in the numbers of cases and deaths was greater during the 28 days preceding 14 September 2025 than the 28 days preceding 16 August 2025.

Many countries decreased surveillance and laboratory testing for COVID-19 when severe illnesses, hospitalizations, and deaths were reduced as a result of the deployment of vaccines and the development of population immunity. The numbers of cases and deaths recorded by the WHO after October 2023 may therefore have been underestimated. The relative recent increase in sequential 28-day records of definitive cases and deaths (Table 1), and their occurrence in countries from different continents, as shown by the WHO COVID-19 data [19], is a cause for concern because it may be due to newly emerging SARS-CoV-2 variants with greater virulence (discussed further in Section 3, Section 4 and Section 5 below).

**Figure 1:**
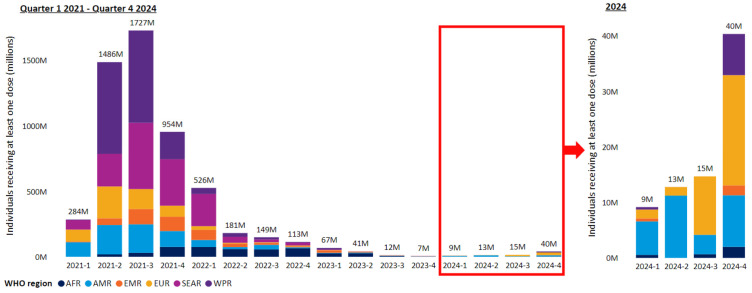
Quarterly vaccination rates for persons receiving at least one vaccine dose across reporting WHO member states by WHO region since beginning of vaccination in the first quarter of 2021 until the fourth quarter of 2024 (reproduced under the creative commons license CC BY 4.0 from reference [19]).

In the first year of COVID-19 vaccination until end December 2021, 55.9% of the global population were estimated to have received one dose of a vaccine, 45.5% to have received two doses, and 4.3% a third booster immunization [27]. By 31 December 2023, this had increased to 67% of the world population receiving a complete first vaccination dose (usually composed of a priming vaccination followed by a recommended second vaccination after a suitable interval) and 32% receiving a booster vaccination after completing the first vaccination [19]. WHO data shows that vaccination rates in many countries were lower in 2024 than the rates achieved from 2021 to 2022 (Figure 1).

**Figure 2:**
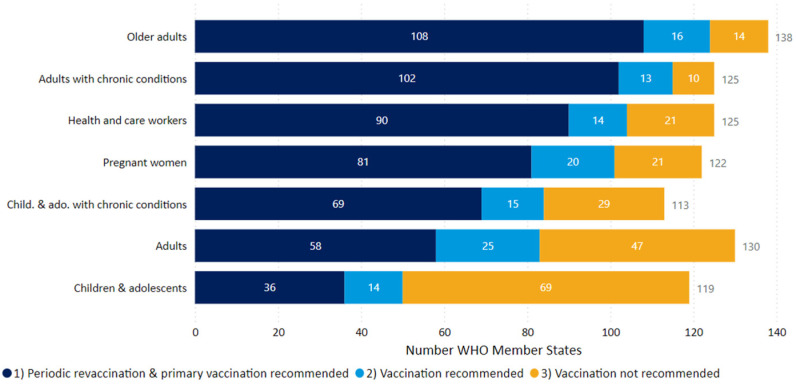
National policies for COVID-19 vaccination for different population groups in WHO member states during 2024. Abbreviations: child—children; ado—adolescents (reproduced under the creative commons license CC BY 4.0 from reference [19]).

WHO member states had established different policies and priorities of vaccinating varying population groups in 2024, as shown in Figure 2. Older adults, adults with chronic health conditions, and healthcare workers were the three groups prioritized for primary vaccination and periodic revaccination in more than 90 WHO member countries [19]. Vaccination was not recommended for healthy children and adolescents in 69 countries.

## 3. SARS-CoV-2 VOI and VUMs in 2025

**Figure 3:**
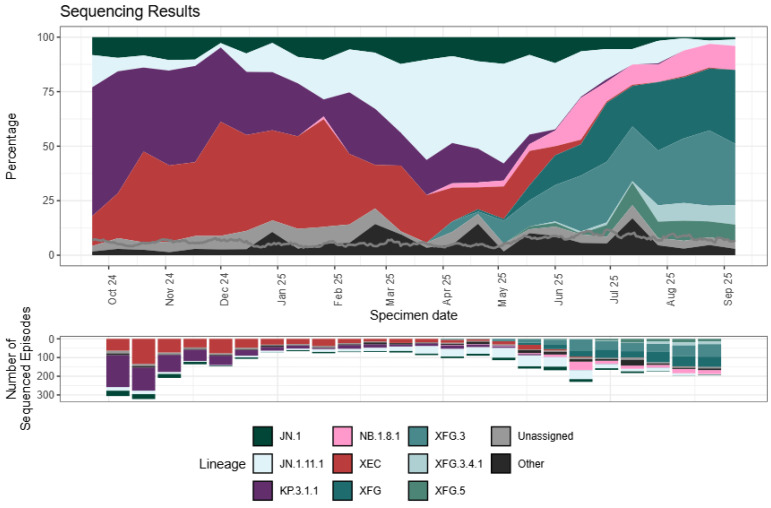
Prevalence of SARS-CoV-2 lineages amongst available sequenced cases for England from 16 September 2024 to 7 September 2025. Reproduced under the creative commons license CC BY 4.0 from reference [42].

The dominant SARS-CoV-2 variant, using the Pango lineage nomenclature [30,38,39,40], at the end of 2023 and in early 2024 in many parts of the world was the Omicron variant JN.1 [10,38], which was designated a VOI by the WHO [19]. JN.1 subsequently evolved into several JN.1 sublineages [10,38]. The JN.1 sublineages and other Omicron lineages in 2025 have given rise to variants that include NB.1.8.1, XFG, LP.8.1.1, and BA.3.2, which have been designated as VUMs by the WHO [38,39]. These variants are characterized by amino acid changes in several SARS-CoV-2 proteins, including S, and the nucleocapsid, membrane, and non-structural proteins [38,41]. As well as unique mutations, convergent mutations that are indicative of selective advantages conferring greater viral fitness were recorded in the evolving variants [41]. The rapid increase in the proportion of the XFG variant lineage sequences globally from April 2025 (Figure 3) has also been observed within the USA [40] and England [42]. The increasing prevalence of XFG and NB.1.8.1 variants justified their classification as VUMs [19,39].

## 4. Assessment of Population Immunity to COVID-19 in 2025

**Figure 4:**
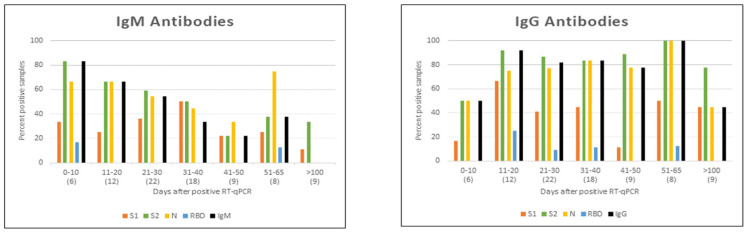
Graphs summarizing IgM and IgG antibody reactivity with recombinant N, S1, S2, and RBD proteins in immunoblots with patient sera obtained at different time intervals after a primary COVID-19 infection confirmed by a positive RT-qPCR test [43]. The graphs show the percentage of sera reacting with each of the four test antigens at different times intervals after the RT-qPCR test. The number of sera tested for each time interval are shown in parentheses in the abscissa. Reactivities with the four SARSCoV-2 proteins are shown in different colors. The black bars show the percentage of sera scored as positive overall for IgM and IgG antibodies with specific diagnostic criteria developed by the testing laboratory. Data reproduced under the creative commons license CC BY 4.0 from reference [43].

The importance of COVID-19 vaccines in eliciting protective immunity that reduced severe illness and mortality during the early stage of the COVID-19 pandemic was highlighted in Section 1.5. Antibodies to the SARS-CoV-2 nucleocapsid (N) protein are produced only after an infection, while antibodies to S are produced both after an infection as well as immunization with S-based vaccines [10]. Experimental data illustrated in Figure 4 show that serum levels of IgG and IgM antibodies to N and S waned markedly by 100 d after a primary COVID-19 infection [43]. The decline in serum antibody levels against S followed a similar time course after the administration of S-based mRNA vaccines [44]. Although serum antibody concentrations decrease with time after infection or vaccination, the memory B and T cells that are also formed by such antigen exposure can be activated by a subsequent infection or vaccination to restore antibody producing and other effector functions [6].

The population-weighted prevalence of antibodies in blood donors aged 17 years and older in England was 95.5% (95% CI 94.6–96.3%) for N and 100.0% (95% CI 99.8–100.0%) for S during the period 20 November 2024 to 24 January 2025 [10]. Only S-based COVID-19 vaccines were almost exclusively used in the UK. The high prevalence of antibodies to N five years after the origin of COVID-19 therefore shows that a very large proportion of the blood donors had experienced SARS-CoV-2 infections by this time. This situation is also likely to apply by extension to the rest of the population in England. It demonstrates that the widespread circulation of SARS-CoV-2 in the population by this time had elicited immune responses in persons who had recovered after COVID-19 infections of varying degrees of severity, or even with no overt symptoms, and with or without prior vaccination. Many people at the present time, i.e., approximately six years after the beginning of the COVID-19 pandemic, will therefore exhibit a combination of infection-induced and vaccine-induced immunity, which is referred to as hybrid immunity.

The clinical protection conferred by natural infection, hybrid immunity, as well as COVID-19 vaccination against asymptomatic infection, symptomatic disease, and severe disease that requires hospitalization and sometimes causes death, decreases with time after the immunity-inducing event [10,11,44,45]. The detailed meta-analysis of data up to 1 January 2023 on the durability of such clinical protection showed that (i) the durability the immunity generated by all three processes waned with protection against severe disease/death being the most durable, (ii) protection against early Omicron variants was substantially lower than against pre-Omicron variants, (iii) boosting vaccinations increased protection against early Omicron variants but without improving durability of immunity, (iv) prior infection increased the durability of vaccine-induced immunity, (v) prior infection with a pre-Omicron variant provided similar initial but more durable protection than primary vaccination against infection with early Omicron variants, and (vi) prior infection enhanced booster-vaccine-induced immunity against early Omicron variants [45].

Specific studies have provided more details of relative protection and the longitudinal decay conferred by different types of exposure to SARS-CoV-2 antigens. One study in the US performed between September 2022 and July 2023 investigated protection against infection, assessed through weekly nasal swabs, in persons who received an S-based mRNA vaccine with or without prior COVID-19 infection [46]. Vaccine efficacy against infection was estimated to be 37.2% (95% CI, 12.3–55.7%) within 7–59 days and 21.1% (95% CI, −0.5% to 37.1%) within 60–179 days of vaccination in previously uninfected persons, and 62.2% (95% CI, 46.0–74.5%) within 7–179 days and 39.4% (95% CI, 12.5–61.6%) at ≥180 days in previously infected persons [46]. Another study in the US modeled the effects of vaccination and prior SARS-CoV-2 infection in protecting against a subsequent infection and severe COVID-19 in the period January 2020 to December 2023 [47]. By 30 December 2023, 98.6% of the U.S. population had been either infected or vaccinated against COVID-19, with 88.3% (95% CI, 78.4–95.5%) being infected at least once [47]. However, the average protection against infection was 31.6% (25.1–41.2%) and against severe disease was 66.1% (59.2–74.3%). The findings also suggested that the newly emergent JN.1 variant may have been responsible for the increase in severe COVID-19 cases observed towards the end of 2023 [47].

It can be concluded that COVID-19 vaccination will continue to be needed at regular intervals, particularly to prevent severe disease, hospitalization, and mortality in population groups with an intrinsically diminished ability to mount an effective immune response [10,42]. These groups include elderly persons, people with chronic illnesses, those undergoing immunosuppressive therapy, and those otherwise immunodeficient, as described in detail elsewhere [7]. Persons in close contact with such vulnerable persons also need to be offered COVID-19 vaccines to minimize the possibility of becoming infected and then transmitting the infection to the more susceptible persons [7]. People with an ancestry in warm tropical climates have been suggested to be more prone to infection with SARS-CoV-2 during temperate zone winters and therefore are more likely to develop more severe COVID-19 [4,5,6], but data from England suggest that their COVID-19 vaccine uptake rate is lower than in other population groups [10,42]. Data from England also show that by the end of the autumn 2024 COVID-19 vaccination campaign, 59.3% of all people aged ≥ 65 y and 23.6% of all people aged <65 y and in a clinical high-risk group had received a boosting mRNA vaccine based on the SARS-CoV-2 variant JN.1 [10], which conferred significant protection against hospitalization due to severe COVID-19 [10]. A similar situation is to be expected in other countries following analogous government policies for vaccination (Figure 2). The policy of vaccinating vulnerable population groups has been continued in England and other countries in 2025 [7,10,19]. The autumn 2025 mRNA vaccines used in England are based on the SARS-CoV-2 variants KP.2 and LP.8.1 [7,48]. These are two of the three SARS-CoV-2 variants, JN.1, KP.2, and LP.8.1, recommended by the European Medicines Agency in May 2025 for use as S-protein-based COVID-19 vaccines in 2025–2026 [49]. COVID vaccines therefore not only need to be administered at regular intervals to boost waning immunity but, like influenza vaccines, require be re-formulated periodically to address antigenic changes in newly emerging SARS-CoV-2 variants.

## 5. Potential of Presently Circulating SARS-CoV-2 Variants to Generate VOCs

Recent analysis of the emerging 2025 variants NB.1.8.1, XFG, LP.8.1.1, and BA.3.2 showed that NB.1.8.1 and XFG had a growth advantage, and BA.3.2 a growth disadvantage compared to LP.8.1.1 [41]. The growth advantage of NB.1.8.1 and XFG were experimentally attributed to better binding of their S protein to ACE2 [41]. However, BA.3.2 showed better evasion of antibodies that inhibit binding between ACE2 and S than NB.1.8.1, XFG, and LP.8.1.1 [41]. These properties have been analyzed in relation to the different mutations in S carried by the four variants [41].

It is also relevant in this context that a monovalent COVID-19 vaccine based on the S of the variant KP.2, which was one type of vaccine recommended for use in 2025–2026 in England [7,48] and Europe [49], elicited serum antibodies with dramatically reduced neutralizing efficiency against the important emerging variants LP.8.1, LP.7.1, NB.1.8.1, XFG, and BA.3.2 [34]. This was attributed to the marked antigenic distance in S between KP.2 and these other variants in addition to antigen imprinting effects from previously encountered S protein immunogens in the KP.2 vaccinees [34]. Such effects can potentially facilitate the possible emergence of VOCs. In general, however, S-based booster vaccines have continued to elicit clinically significant protective immunity against COVID-19 [44,45,46,47].

Protective adaptive immune responses against infection with SARS-CoV-2 depends not only on antibodies but also on T cells in the URT and its associated nasopharynx-associated lymphoid tissue (NALT) [6,20]. This was first experimentally demonstrated in healthcare workers who remained seronegative and therefore uninfected with SARS-CoV-2, in spite of repeated contact with COVID-19 patients during the early stage of the COVID-19 pandemic [50]. This protection was attributed to the T cells that recognized cross-reacting epitopes in proteins other than S from commonly circulating coronaviruses that cause cold-like symptoms [50]. Other evidence supporting important roles for memory CD4 T helper and CD8 T cytotoxic cells in the NALT in protecting the URT against fresh SARS-CoV-2 infections have been previously reviewed [5,6,20].

The 29 proteins encoded by the SARS-CoV-2 genome include 4 structural, 16 non-structural and 9 accessory proteins [51]. The effect of mutations in S on viral fitness, notably the evasion of invasion-inhibiting antibodies, has been well documented [6,24,41,51]. Limited evidence suggests that mutations in S and several other SARS-CoV-2 proteins are also involved in evading T cell-mediated cellular immune responses [6,36,51]. The possible roles of different SARS-CoV-2 proteins in influencing innate immune responses as well as other aspects of viral fitness such as replication rates were recently reviewed [51]. A specific example pertinent to escaping innate immunity is the methionine-to-isoleucine substitution in a peptide that binds HLA-E in the Omicron BQ.1 variant and its sublineages, which interferes with the killing of virus-infected cells by natural killer (NK) cells [52]. The monitoring of mutations in other SARS-CoV-2 proteins besides S in emerging variants is therefore also necessary.

SARS-CoV-2 infections had been identified in 29 species of domestic, farmed, and wild animals in 36 countries up to the end of 2023 [53]. The virus became established in wild white deer populations in the US and also in farmed mink, which were able to transmit the infection back to humans [53]. The Omicron variant JN.1 has been shown to more efficiently utilize ACE2 from different species of domestic and wild animals for the invasion of cells in vitro [35]. Variants that arose form JN.1, and that are dominant in 2025, may also exhibit this property but this requires experimental verification. SARS-CoV-2 variants with Pango names that begin with X have arisen through recombination occurring between different virus strains within human cells, e.g., XFG, which is a recombinant of the LF.7 and LP.8.1.2 lineages and a current VUM [38]. SARS-CoV-2 can be expected to accumulate novel mutations when it adapts to infect, replicate, and transmit in animal populations, which in turn can generate variants possessing complex genetic reassortments and new abilities to infect and proliferate in humans. Novel SARS-CoV-2 VOCs can potentially arise in this way. The swine origin of the 2009 H1N1 influenza A pandemic provides a precedent for this process [54].

The characteristics of important past VOCs and recent Omicron variants summarized in Table 2 illustrate how selection factors influencing the spread and prevalence of SARS-CoV-2 variants changed during the different phases of the COVID-19. It shows essentially that greater transmissibility, due to factors such as infectibility, virus replication rate, and avoidance of early innate immune responses, was gradually replaced or supplemented by the ability to evade neutralizing antibodies, which resulted from increasing population immunity.

## 6. Outlook and Strategies for COVID-19 Control in the Future

There was optimism during the early stages of the COVID-19 pandemic that a high enough prevalence of persons who had developed immunity through infection, vaccination or both can reduce new infections of SARS-CoV-2 sufficiently to eliminate COVID-19, i.e., herd immunity can be achieved against COVID-19 [23]. Herd immunity, largely attained by the use of a highly efficacious vaccine, was primarily responsible for the eradication of smallpox in 1980. There is now a general realization that it is not possible replicate this success with COVID-19 because of a combination of the following factors: (i) higher rates of person-to-person transmission of SARS-CoV-2, (ii) rapid selection of mutations that lead to evasion of immunity, (iii) insufficient efficacy of presently available COVID-19 vaccines to prevent infection although they reduce severe disease and mortality, (iv) relatively rapid waning of immunity against SARS-CoV-2, and (v) the levels of social distancing, national and international travel restrictions, and other non-pharmaceutical interventions that helped reduce SARS-CoV-2 transmission early in the pandemic are no longer socially or economically acceptable [7,10,23,31,45,90,91].

The presently circulating Omicron variants, helped by prevalent immunity levels, are causing less severe disease and mortality in young healthy people than the earlier VOCs like Alpha and Delta [12,13,30]. Therefore, the present high levels of immunity in the population appear to be driving SARS-CoV-2 to adopt characteristics of the disease caused by the other human coronaviruses that produce common cold-like infections in the URT [88]. Many COVID-19 vaccines at the present time that are largely based on intramuscular immunization with S need to be reformulated annually with the S antigens from the dominant circulating SARS-CoV-2 variants, directed towards generating neutralizing anti-S antibodies, and administered particularly to the more vulnerable population groups [19,42,48,49]. Influenza vaccines in use at present, that are also reformulated annually to address antigenic variation in the influenza virus, are available as recombinant proteins for the intramuscular vaccination of adults and the elderly, and as a live attenuated virus vaccine for intranasal administration to 2–18 y old children [92]. Besides antibodies, effector CD4 T and CD8 T cells have multiple roles in protecting against COVID-19, including (i) CD4 T cell-mediated activation of B cells to become antibody-producing plasma cells, and (ii) CD4 T and CD8 T cells responding to epitopes in multiple SARS-CoV-2 proteins including S, N, membrane, and non-structural proteins, controlling infection and reducing COVID-19 severity [6,20,93]. SARS-CoV-2-specific CD4 T and CD8 T cells are detectable in the lymphoid tissues of the URT, LRT, and elsewhere in the body after an infection, and these include memory CD4 T and CD8 T cells present long after SARS-CoV-2 infections, including asymptomatic infections [6,20,93]. Furthermore, (i) non-S proteins have more conserved sequences, and thereby more conserved T cell epitopes than S between SARS-CoV-2 variants, (ii) T cell responses are largely preserved between Omicron variants, and (iii) the breadth of T cell responses makes it difficult for SARS-CoV-2 variants to avoid T cell-mediated immunity [6,20,93]. Therefore, vaccines that promote T cell responses against multiple SARS-CoV-2 proteins, as well neutralizing antibodies against S, and that are delivered to induce URT as well as systemic immunity, may provide better protection against COVID-19. Vaccines based on live attenuated SARS-CoV-2 can elicit T cell responses and antibodies against multiple virus proteins including S and have shown promising results in preclinical studies [94,95,96]. Since intranasal mucosal immunization, is optimal for inducing protective innate and adaptive immunity in the URT [6,20,24,25,26], the finding that a parainfluenza-virus-vectored vaccine expressing S administered intranasally was safe, elicited serum and mucosal antibodies to S, and afforded significant protection against symptomatic COVID-19 demonstrated an encouraging path forward [97]. Similarly, an adenovirus-vectored vaccine expressing the S1 subunit of S, N, and RNA-dependent RNA polymerase, administered by inhalation, has been shown to produce trained innate immune responses in alveolar macrophages, antibodies, and T cell-mediated immune responses in the lung [98].

Current late-autumn 2025 health surveillance data from the UK, which may be representative of northern hemisphere temperate zone countries, suggests that COVID-19 was less of a problem than influenza, with approximately a 50% lower rate of admission into hospital and intensive care units [99]. However, the continuing generation of new SARS-CoV-2 variants and the rapid global spread of the more fit variants highlight the need to continue the worldwide effort to determine their genome sequences and virological characteristics in order to effectively control COVID-19. Appropriate monitoring of emerging SARS-CoV-2 variants in animals also seems prudent. Besides sequencing variant viruses from infections, the sequencing of SARS-CoV-2 obtained through monitoring wastewater can be a useful adjunct method that can be applied to pertinent locations like airports and farms [100,101,102]. In addition to monitoring emerging variants for their ability to evade neutralizing antibodies against S and changes in growth characteristics as is commonly performed, identifying transmissible mutations showing increased drug resistance, notably in immunocompromised patients [30,103], and reducing the recognition of S and other virus proteins by helper T and cytotoxic T cells [36,104] as well as mutations in virus proteins that increase virus fitness in further ways, for example, through reducing innate immune responses [51,52], are also important.

## Figures and Tables

**Table 1:** WHO recorded cumulative cases and deaths due to COVID-19 on 14 September 2025 [19].

Location	Cumulative Cases Until 14 September 2025	Cases in the 28 d Preceding 14 September 2025	Cases in the 28 d Preceding 16 August 2025	Cumulative Deaths Until 14 September 2025	Deaths in the 28 d Preceding 14 September 2025	Deaths in the 28 d Preceding 16 August 2025
Worldwide	778.7 million	132,801	79,253	7.1 million	1671	1187

**Table 2:** Features of past and present prominent SARS-CoV-2 variants.

COVID-19 Phase	Variant and Its Origin and Prevalence	Mutations	Immune Evasion	Transmissibility	Disease Severity
Before vaccination	Variant with D614G mutation in S. Multiple global origins around March 2020 [38,55]. Evolved into later VOCs [56].	D614G in S and linked mutation in RNA polymerase [55,56,57,58,59].	Not seen with convalescent serum antibodies [57,58,59].	Greater than the ancestral Wuhan strain [57,58,59].	Marginally greater than the ancestral strain [57,58].
	Alpha VOC, B.1.1.7 Originated in UK mid-2020 and then spread worldwide, but is no longer in circulation [38].	In total, 17 mutations in S including D614G, N501Y, ΔH69-ΔV70, and P681H [56,60,61,62].	Small neutralization reduction with ancestral S mRNA vaccine-elicited sera compared with ancestral strain [63].	Increased compared to earlier strains [60,61,62].	More severe than ancestral strain [60,61,62].
Early post-vaccination	Delta VOC, B.1.617.2 Originated in India in April 2021 and then spread widely but is no longer in circulation [38,64].	Additional mutations in S including P681R, L452R, and others in RNA polymerase and additional viral proteins [65,66,67,68,69].	Markedly reduced sensitivity to immune antibodies [70,71].	Increased compared to Alpha [65].	More severe than Alpha [68,69].
After large-scale vaccination and COVID-19 infection	Omicron B.1.1.529 VOC first identified in Africa in November 2021 [72], and the precursor for other Omicron variants	Increasing number of mutations compared to earlier VOCs in S, notably in RBD, and also in other viral proteins [73,74,75,76,77].	Evasion of antibodies in convalescent and vaccinee sera, and monoclonal antibodies used in therapy [75,76,77,78,79].	Changes in intrinsic transmissibility unclear [73].	Less than Delta and earlier VOCs [73,74,80,81,82,83,84,85,86].
	Omicron JN.1 first characterized in August 2023 and designated VOI in December 2023 [87]. Largely replaced in 2025 by more fit offspring variants, e.g., XFG (Figure 3) [41,42].	More mutations in S compared to earlier Omicrons, notably L555S [35,41,87].	Greater evasion of invasion inhibiting antibodies [35,36,87], and possibly cytotoxic T cells recognizing S and N [36].	More transmissible than earlier Omicrons to other persons and also animals [35].	Greater transmissibility and infectibility causing more infections [35,36,47,87]. Evidence for preferential URT replication and lower LRT pathology than earlier VOCs [88].
	Omicron XFG first identified in January 2025. Designated a VUM. XFG and its sublineages dominant globally in late 2025 [89].	XFG has 11 additional mutations in S compared to JN.1 [89].	Available data suggest greater evasion of neutralizing antibodies compared to LP.8.1.1 [41].	High relative growth advantage compared to co-circulating variants [41].	No evidence for greater disease severity than other co-circulating Omicron variants [89].

## Data Availability

All data necessary for interpretation are included in the manuscript or publicly available citations.
